# Experimental Infection of Dogs with Leishmania and Saliva as a Model to Study Canine Visceral Leishmaniasis

**DOI:** 10.1371/journal.pone.0060535

**Published:** 2013-04-05

**Authors:** Dirceu Joaquim Costa, Rayssa M. de Araujo Carvalho, Melissa Abbehusen, Clarissa Teixeira, Maiana Pitombo, Joelma Trigo, Flávia Nascimento, Lucilene Amorim, Ana Lucia Abreu-Silva, Maria do Socorro Pires Cruz, José Carlos Miranda, Kyoshi Fukutani, Camila I. de Oliveira, Aldina Barral, Manoel Barral-Netto, Cláudia Brodskyn

**Affiliations:** 1 Centro de Pesquisa Gonçalo Moniz, FIOCRUZ-BA, Bahia, Brazil; 2 Faculdade de Medicina, Universidade Federal da Bahia, Bahia, Brazil; 3 Instituto de Ciências da Saúde, Universidade Federal da Bahia, Bahia, Brazil; 4 Laboratório de Imunofiosiologia, Departamento de Patologia Universidade Federal do Maranhão, Maranhão, Brazil; 5 Departamento de Patologia, Universidade Estadual do Maranhão, Maranhão, Brazil; 6 Departamento de Morfofisiologia Veterinária Centro de Ciências Agrárias, Universidade Federal do Piauí, Piauí, Brazil; 7 Instituto de Investigação em Imunologia, São Paulo, Brazil; INRS - Institut Armand Frappier, Canada

## Abstract

**Background:**

Canine Visceral Leishmaniasis (CVL) is a zoonotic disease caused by *Leishmania infantum*, transmitted by the bite of *Lutzomyia longipalpis* sand flies. Dogs are the main domestic reservoir of the parasite. The establishment of an experimental model that partially reproduces natural infection in dogs is very important to test vaccine candidates, mainly regarding those that use salivary proteins from the vector and new therapeutical approaches.

**Methodology/Principal Findings:**

In this report, we describe an experimental infection in dogs, using intradermal injection of *Leishmania infantum* plus salivary gland homogenate (SGH) of *Lutzomyia longipalpis*. Thirty-five dogs were infected with 1×10^7^ parasites combined with five pairs of *Lutzomyia longipalpis* salivary glands and followed for 450 days after infection and clinical, immunological and parasitological parameters were evaluated. Two hundred and ten days after infection we observed that 31,4% of dogs did not display detectable levels of anti-*Leishmania* antibodies but all presented different numbers of parasites in the lymph nodes. Animals with a positive xenodiagnosis had at least 3,35×10^5^ parasites in their lymph nodes. An increase of IFN-γ and IL-10 levels was detected during infection. Twenty two percent of dogs developed symptoms of CVL during infection.

**Conclusion:**

The infection model described here shows some degree of similarity when compared with naturally infected dogs opening new perspectives for the study of CVL using an experimental model that employs the combination of parasites and sand fly saliva both present during natural transmission.

## Introduction

Canine visceral leishmaniasis (CVL) is caused by an intracellular protozoan parasite *Leishmania infantum.* It is endemic in the Mediterranean Basin, South America and parts of Asia [1]. Domestic dogs are the main reservoirs and different control strategies, such as the use of insecticide impregnated collars or elimination of infected dogs have not been effective to decrease human incidence of VL [2]. Development of a vaccine for CVL has been identified as a research priority by WHO/TDR [3] and mathematical models have highlighted canine vaccination as the potentially most practical and effective means of impacting disease control in humans [4]. Also, since dogs present many symptoms observed in humans, with a long period of asymptomatic infection followed by wasting, anaemia, enlarged lymph nodes, and fever, the canine model is important to study VL pathogenesis and for development of pre clinical trials related to therapy. Although an experimental canine model for VL is highly desirable previous attempts to infect dogs have used the inoculation of a high number of parasites intravenously that in some occasions did not result in disease development [5], [6], [7].

*Leishmania* parasites are transmitted by female sand flies that co-inject parasites and different products from the vector, including saliva, in the host’s skin. Saliva of sand flies and of other blood feeding arthropods contains potent pharmacological components to facilitate the blood meal. Salivary proteins also play an important role during pathogen transmission as co-inoculation of sand fly saliva with the parasite exacerbates parasite infectivity [8], [9], [10], [11], [12], [13], [14], [15]. Although the use of vector saliva and Leishmania in different experimental models such as mice and hamsters have been employed, few studies used this experimental approach in dogs and results are divergent [16], [17] Therefore, the establishment of an experimental model of infection in dogs, using parasites and saliva, could be very important in the context of *Leishmania* natural transmission.

Such model would therefore be useful to test new approaches of vaccines against CVL and our present research line is to test potential vaccine candidates employing salivary proteins from the vector.

Herein, we report that the use of stationary phase promastigotes of *Leishmania infantum* and salivary gland homogenate (SGH) of *Lutzomyia longipalpis* results in disease development in 100% of the dogs with different degrees of disease severity. Besides that, comparing experimentally and naturally infected dogs we noticed that clinical symptoms as well as inflammatory responses were very similar suggesting that the currently developed model is appropriate for our future objectives, which will test vaccine candidates using salivary proteins.

## Materials and Methods

### Animals

In this study, we used 35 experimentally infected and eight naturally infected dogs. We purchased thirty-five beagles of both genders (eight to ten months old), in a non-endemic area from Brazil, from a local breeder (Canil Tad’s Henriques, Colombo, Paraná State, Brazil). All procedures performed in experimentally infected dogs were approved and permitted by the Ethical Committee for Animals Use (CEUA) from Centro de Pesquisa Gonçalo Moniz/Bahia - FIOCRUZ/Ba, under the number 010/2009. The study was supported by the Financial Agency from Estado da Bahia (FAPESB). After quarantine, all dogs received routine vaccinations and had negative anti-*Leishmania* and anti-saliva (*Lutzomyia longipalpis*) antibody tests. The animals were housed at the Experimentation Kennel facility in Monte Gordo (Camaçari, Bahia State, Brazil). Eight naturally infected mongrel dogs of both genders and different ages were obtained at Teresina (Piauí State, Brazil) where the incidence of CVL is high (3,429 infected dogs in a total population of 18,661) [18]. These naturally infected dogs were obtained at the Center of Zoonosis Control (CZC) of Piauí that receive all Leishmania-infected dogs for euthanasia, since in Brazil according to the law of the Ministry of Health, all dogs that are positive for Leishmania by detection of specific antibodies, using ELISA or indirect immunofluorescence, must be euthanized. Therefore, these eight dogs’ samples were obtained at CZC and their use was also approved by Centro de Pesquisa Gonçalo Moniz/Bahia- FIOCRUZ/Ba, under the number 0429/07.

During the experiments, dog stress was minimized by anesthesia, facilitating the animal manipulation and reducing the time of procedures. After performing the experiments the animals were followed every day to verify possible inflammatory reactions at the site of manipulation and in the case of pain after clinical exam, we used analgesic drugs orally. In the case of a persistent pain, presence of local edema or even if the animal presented problems to walk; we used an anti-inflammatory drug, in a therapeuthical dose, once a day, orally for three days.

The animals were submitted to euthanasia when they presented severe symptoms that characterize Canine Visceral Leishmaniasis. The animals were euthanized by intravenous injection of anesthesia composed of the association of acepromazin/ketamin, in a higher dose than usually employed for anesthesia. After injection, death was confirmed by cardiac respiratory arrest. All dogs were euthanized at the end of the experiments according to the recommendation of the Ministry of Health. Following euthanasia, skin samples were processed to investigate the presence of parasites by immunohistochemistry.

### Sand Flies and SGH Preparation

*Lutzomyia longipalpis*, Cavunge strain (Cavunge, Bahia) was reared at the Laboratório de Imunoparasitologia/CPqGM as previously described [19]. Salivary glands were dissected from five- to seven day old females and stored in saline at -70°C. Before use, salivary glands were sonicated and centrifuged at 8.000 g for five minutes. The supernatant was collected and used immediately.

### Intradermal Infection

For experimental infection, *Leishmania infantum* (MCAN/BR/00/BA262) promastigotes were cultured in Schneider’s medium (LGC, Brazil) supplemented with 10% of inactivated FBS (fetal bovine serum), 2 mM L-glutamine, 100 IU/ml penicillin, 1% streptomycin. Dogs were inoculated by intradermal route, in the ear with 10^7^ stationary phase promastigotes in the presence of SGH equivalent to five pairs of glands using a 29-gauge needle in a volume of 200 µl.

### Clinical Evaluation

An independent veterinarian carried clinical examinations of the dogs monthly after infection looking for signs and symptoms of CVL. The degree of CVL was defined according to signs such as nutritional state (loss and variation of weight), skin involvement, lymphadenomegaly, conjunctivitis, size of nails (onychogryphosis) and splenomegaly that were assigned a score from 0 to 2 at each time point, adapted from Manna *et al.* [20]. At the end of evaluation a sum of points was obtained and this value was considered the clinical score of each dog. The same parameters were applied to naturally infected dogs and a score was attributed to each animal.

### DNA Extraction and Parasite Burden Quantification by Real-time PCR

In experimental infected dogs, after 240 and 450 days of challenge, popliteal lymph nodes (LN) biopsies and PBMC were harvested and homogenized in 500 µL of extraction buffer (0,01 M Tris-HCl, 0,001% EDTA, 0,02% SDS, 8 M urea, 0,3 M NaCl). Later, 500 µL of saturated phenol:chloroform:isoamilic alcohol solution (25∶24:1) was added, homogenized and centrifuged at 13,000 rpm for five minutes. Supernatants were harvested after new homogenization and centrifuged with 500 µL of saturated phenol:chloroform:isoamilic alcohol solution. Ammonium acetate at 2 M (45 mL) was added and the volume was completed to 1.5 mL with cold absolute ethanol. Pellets were washed with cold ethanol 70%, supernatants discarded and DNA dried at room temperature. After drying, DNA was eluted in 20 µL of water at 55°C for 10 minutes and stored at −70°C until use. To determine parasite loads in lymph nodes and PBMC in experimentally infected dogs, Real-Time PCR using 100 ng of DNA was performed. Samples were amplified with the ABI Prism 7500 Sequence Detection System using real-time SYBR-Green PCR master mix kit (Applied Biosystems) and 250 nM of internal probes 23F: 5′-TCCCAAACTTTTCTGGTCCT-3′ and 154R: 5′-TTACACCAACCCCCAGTTTC-3′ (Gene Bank Identification Z35273.1), that target *Leishmania* kinetoplast DNA [21]. Amplification conditions consisted of an initial pre-incubation at 95°C for 10 min, followed by amplification of the target DNA for 40 cycles of 15 seconds at 95°C and 1 minute at 60°C with the ABI Prism 7500 Sequence Detection System (The Perkin-Elmer Corporation, USA), according to the manufacturer’s manual. A standard curve was generated by amplification of *Leishmania infantum* promastigotes DNA, whose first point was 140 ng of DNA (equivalent to 10^9^ parasites) serially diluted to 1.4 fentograms (equivalent to 10^1^ parasites) per microliter.

### Immunohistochemistry

Immunohistochemistry of skin sections of infected dogs was performed to verify the presence of parasites. The assay used the streptavidin peroxidase reaction, which was carried out in accordance with Tafuri *et al.* [22] in paraffin-embedded segment tissue samples. Briefly, skin samples were fixed in 10% formalin and embedded in paraffin. Sections (5-µm thick) were taken on poly-L-lysine coated slides for immunohistochemistry. The anti-sera against *Leishmania* were obtained from rabbits chronically infected with Leishmania *infantum* and were applied on sections at dilution 1∶1,000. Staining was done employing Dako Advanced HRP kit (Dako, Glostrup, Denmark). Isotype control antibody (R&D Systems) was used as negative controls. Five fields were counted for each slide by two independent observers to perform parasite quantification.

### Xenodiagnosis

To assess whether infected dogs were able to transmit parasites to the vector, xenodiagnosis was performed 450 days after infection. Thirty *Lutzomyia longipalpis* female sand flies, between five to seven day old were transferred to a feeder (50 cm diameter×5 cm height) with an open side covered by a fine nylon mesh placed over the skin on the internal ear surface and covered with a piece of black fabric for 40 minutes. Sand flies were transferred to plastic pots with plaster at the base, where they were kept for five to seven days before dissection. Dissected midguts were checked under light microscope for the presence of promastigotes. Genomic DNA was extracted from the midguts of sand flies that resulted negative by microscopic visualization to be analyzed by conventional PCR for *Leishmania infantum* using the primers 150 (5′ TGGGGGAGGGGCGTTCT 3′) and 152 (3′ AACTGGGGGTTGGTGTAAATT 5′) [23]. PCR products were analyzed by electrophoresis in a 1% agarose gel stained with ethidium bromide.

### Soluble *Leishmania* Antigens (SLA) Preparation

*Leishmania infantum* SLA was prepared as previously described [24] with modifications. Briefly, promastigotes were washed six times in sterile cold 0.8% PBS (pH 7.2) supplemented with 1% glucose at 3,000 g for 20 min. Leupeptin at 1% and 2 mM EDTA were added to the pellet after the last wash. The pellet was resuspended in five ml of sterile water and freeze-thawed 10 to 12 times. The suspension was sonicated, on ice, at 40 hertz, three times, using a sonic disrupter (Sonifier 250 - Branson Ultrasonics Corporation, USA). The volume was adjusted to 10–20 mL in sterile PBS and centrifuged at 12,000 g for 30 minutes. The supernatant was then harvested, filtered (0.2 µm) and protein concentration determined by the Bio-Rad Assay kit (Bio-Rad, Hercules, CA), based on a bovine serum albumin (BSA) standard curve as previously described [25] and stored at −70°C until use.

### Humoral Immune Response

The levels of antibodies anti-*Leishmania* during infection were determined by conventional enzyme-linked immunosorbent assays (ELISA), using SLA. Serum samples were added at dilutions of 1∶400. Following washing, a rabbit anti-dog IgG alkaline phosphatase conjugate (1∶4,000, Sigma, Missouri, USA) was added and incubated for one hour. The wells were then re-washed, substrate and chromogen (p-nitrophenyl phosphate; Sigma, USA) were added, and absorbance was recorded at 450 nm on a SpectraMax 190 spectrophotometer (Molecular Devices, USA) automatic microplate reader.

### Cell Culture and Cytokine Detection

At day zero, 90, 180, 270 days after infection, PBMC were isolated from 10 mL samples of heparinized blood, which was layered onto three mL of Ficoll-Paque PLUS density gradient 1.077 (GE Healthcare) and centrifuged at 450 g for 30 min at room temperature. Cells were then washed three times in saline and finally resuspended in RPMI 1640 medium supplemented with two mM L-glutamine, penicillin (100 U/ml), gentamicin (100 µg/ml), and 10% heat-inactivated fetal bovine serum (HyClone) at 5×10^6^ cells/mL. We used a final volume of one ml per well in the cultures. Cells were then incubated with live stationary phase *Leishmania infantum* promastigotes obtained from Schneider’s culture (ratio parasite:cell 1∶1) or concanavalin A at a final concentration of five µg/mL, at 37°C, 5% CO_2_ for 24 or 48 h. Supernatants were harvested after 48 and 72 hours and kept at −20°C until assayed for determination of cytokine levels. Levels of IL-10 in supernatants were determined by ELISA (assay range from 31.2 to 2,000 pg/ml) using commercial anti-cytokine antibody pairs (R&D Systems, USA) according to manufacturer instructions, IFN-γ was determined by ELISA using commercial Quantikyne Immunoassay (assay range from 62.5 to 4,000 pg/ml) (R&D Systems, USA).

### Statistical Analysis

Statistical analysis was performed using GraphPad Prism 5.0 program (GraphPad Software, USA). Comparisons of cytokine (pre vs. post stimulation) or IgG levels evaluated during infection were performed using the paired and non-parametric Wilcoxon test. Correlation was performed using nonparametric Spearman test and linear regression parameter. Sensitivity of xenodiagnosis was calculated by comparing positive sand flies and parasite loads in lymph nodes and PBMC using the ROC (Receiver Operator Characteristic) curve taking into account the area under the curve and the parasite load cut-off displaying the highest likelihood ratio, sensitivity and specificity. Evaluation of immunohistochemistry morphometry was performed using non-parametric Mann Whitney test. Differences were considered significant when P values ≤0.05.

## Results

### Clinical Evaluation of Experimentally Infected Dogs

We infected the dogs using intradermal route with 10^7^ stationary promastigotes of *Leishmania infantum* plus five pairs of salivary glands of *Lutzomyia longipalpis.* The amount of salivary glands utilized in this study is higher compared to others, since we also increased the number of parasites and in order to obtain an effect as described in literature about saliva and establishment of infection, we calculated five pairs of salivary glands as proportional to the number of parasites used.

**Figure 1:**
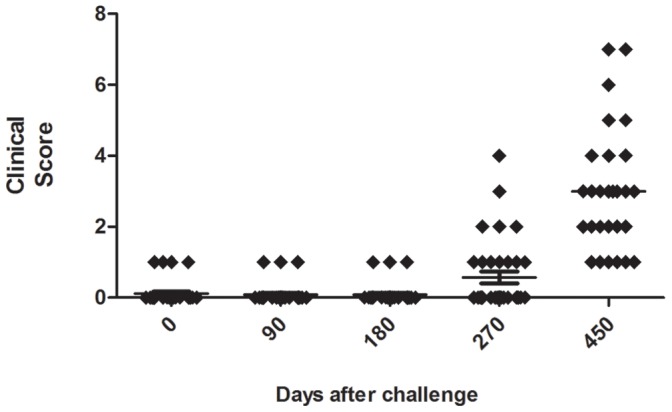
Clinical evaluation of infected dogs. Dog were intradermally infected with 10^7^ parasites plus five pairs of *Lutzomyia longipalpis* salivary glands, through the infection (day zero to day 450 post infection) according the parameters described in Material and Methods section. The sum of parameters resulted in the clinical score shown in the figure.

Clinical assessment of dogs was performed by evaluating the severity of clinical signs of *Leishmania infantum* infection. There was an increase in the frequency of dogs displaying severe symptoms during infection, and 450 days after infection, 14% of dogs showed clinical score up to seven (Figure 1). The most common signs observed in these dogs were onycogryphosis and lymphadenopathy (78.6%), fur changes (50%) and splenomegaly (14%). According our protocol approved by the Ethical Committee in Use of Animals, dogs presenting severe disease, with a high clinical score must be euthanized. Therefore, over the period of observation, 20% (7/35) of the animals showed severe symptoms and were euthanized 240 days after infection.

Naturally infected dogs had a similar score, around 9.5, and the most frequent clinical signs included lymphadenopathy and splenomegaly (100%), fur changes (77%), nutritional status and weight loss (67%).

### Parasitological Parameters

**Figure 2:**
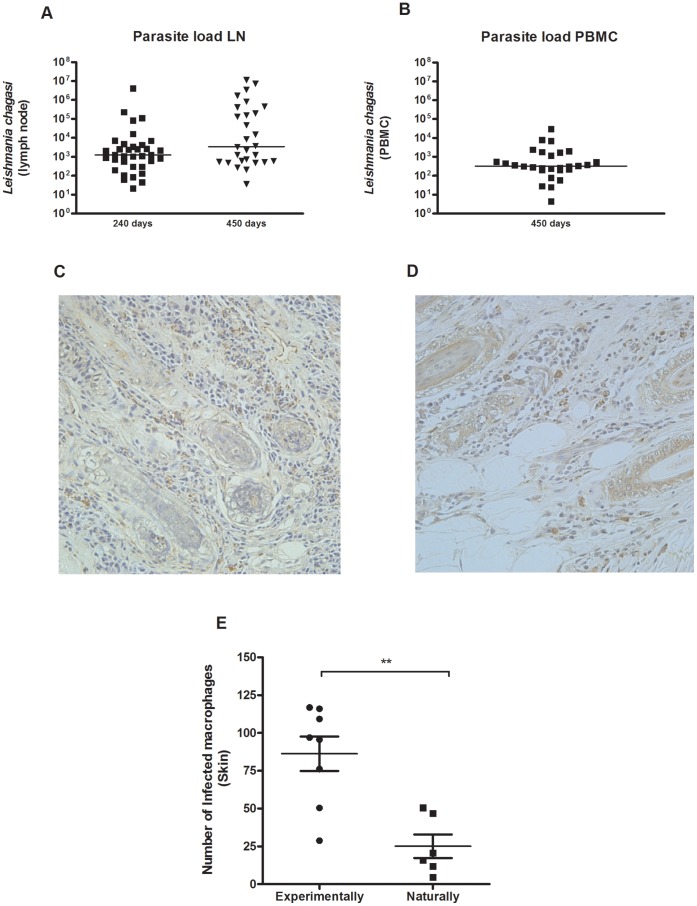
Parasitological parameters in experimentally and naturally infected dogs. Parasite load in dogs experimentally infected with 10^7^ promastigotes of *Leishmania infantum-chagasi* plus five pair of *Lutzomyia longipalpis* salivary glands through intradermal route. Total DNA was extracted from popliteal lymph node aspirate and used for *Leshmania infantum chagasi* detection by real time PCR 240 and 450 days after infection (A). Parasite load in PBMC from infected dogs 450 days after infection (B). Skin sections from experimentally infected dogs (C) and naturally infected dogs (D) (400x). Number of parasites in the skin of experimentally and naturally infected dogs by immunohistochemistry and quantified by morphometry (E).

Popliteal lymph nodes aspirate was obtained to measure parasite load by Real-time PCR, using specific primers for *Leishmania infantum.* Although we detected variation in the number of parasites found in these organs, parasites were detected in all dogs 240 days after infection. Parasites were still detected 450 days post-infection with a slight increase comparing to 240 days post-infection (Figure 2A). Interestingly, we did not detect any parasites at 240 days after infection in the PBMC, but at the time point of 450 days post infection parasites were detected in this compartment (Figure 2B), but the number observed is lower than those obtained at lymph node aspirates (median of 10^2^ parasites/10^6^ PBMC). We obtained eight naturally infected dogs at Piauí, and they were euthanized by Center of Zoonosis Control (CZC). Unfortunately, we did not have access to the popliteal lymph nodes or PBMC samples from these dogs. We only obtained skin biopsies of these animals, besides clinical evaluation at the time of euthanasia. Therefore, in order to compare some aspects between naturally and experimentally infected dogs, we evaluated the presence of parasites in the skin by immunohistochemistry that were quantified by morfometry (Figure 2C and D). Experimentally infected dogs presented a significantly higher number of infected cells than naturally infected dogs (Figure 2E).

### Transmissibility to Sand Flies

**Figure 3:**
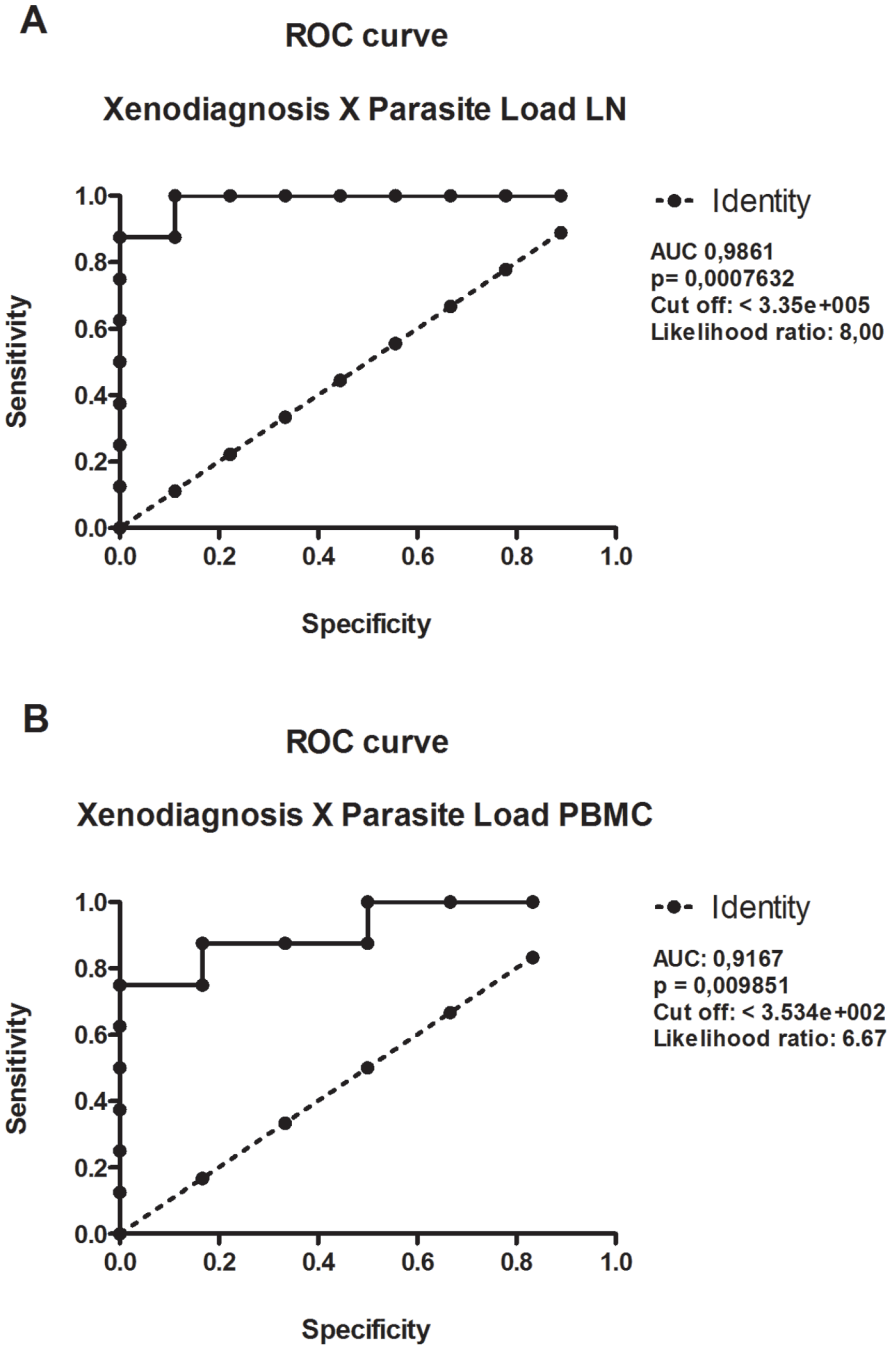
ROC curve of parasite load threshold levels predicting xenodiagnosis positivity. A ROC curve was built using data regarding parasite load levels in lymph nodes (A) or PBMC (B) against the results of xenodiagnosis from 30 dogs infected with *Leishmania infantum-chagasi* plus five pairs of *Lutzomyia longipalpis* salivary glands, 450 days post infection.

We evaluated the ability of experimentally infected dogs to transmit parasites to sand flies through xenodiagnosis. Each dog was exposed to 30 female sand flies that were allowed to feed for 40 minutes. Five to seven days after blood meal sand flies midguts were individually dissected to check the presence of promastigotes. Sand flies that were negative by microscopic visualization were analyzed by PCR using specific primers for *Leishmania infantum* to increase the sensitivity of detection. We found that 16.66% and 10.71% (total of positive xenodiagnosis 27.37%) of infected dogs presented a positive xenodiagnosis, by light microscopy and PCR, respectively. Based on these results and lymph node aspirates and PBMC parasite loads, we constructed a ROC curve to determine the cut-off values of parasite burden necessary to provide a positive result in the xenodiagnosis assay. As shown in Figure 3, the minimum number of parasites to obtain a positive xenodiagnosis is 3.35×10^5^ parasites (p<0,0008) in the lymph nodes and 3.53×10^2^ parasites in the PBMC (p<0,001).

### Detection of Anti-*Leishmania* Antibodies

**Figure 4:**
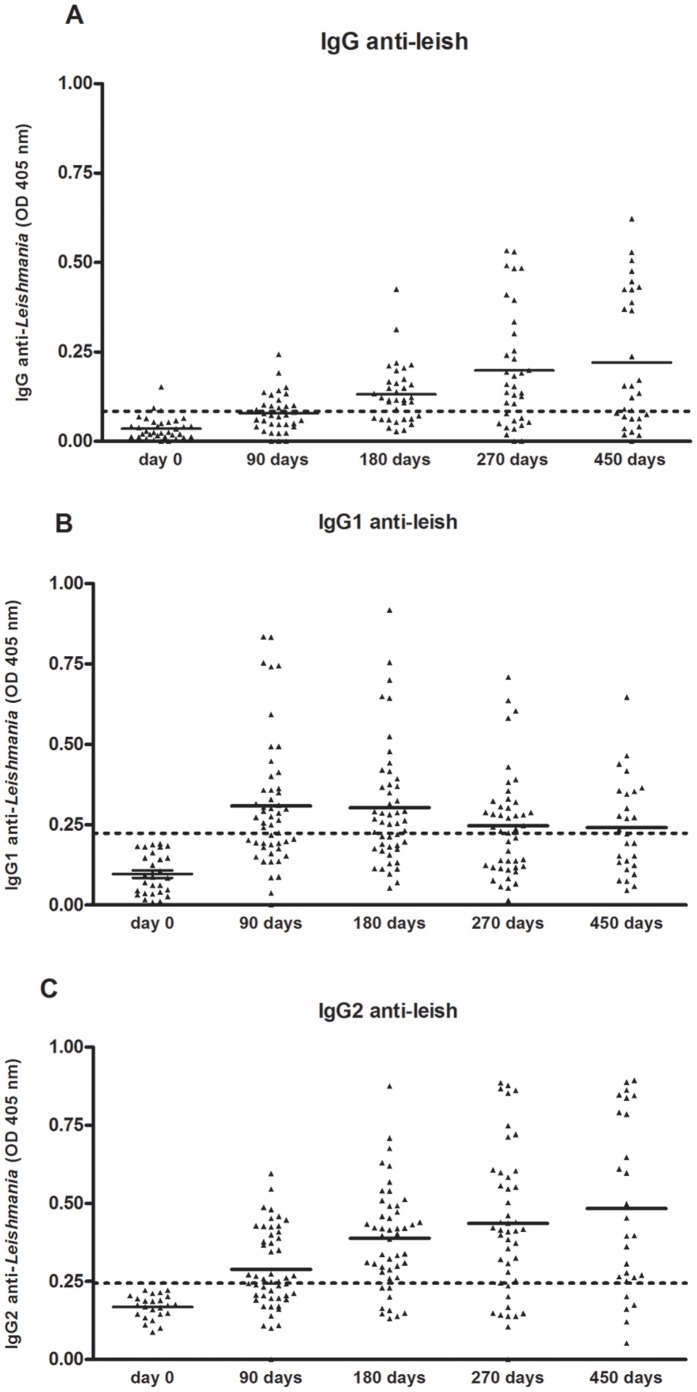
Serological parameters in experimentally infected dogs. IgG (A), IgG1 (B) and IgG2 (C) antibody levels determined by ELISA in dogs experimentally infected with *Leishmania infantum-chagasi* plus five pairs of *Lutzomyia longipalpis* salivary glands. Dotted line indicates cut-off point established at 2SD above uninfected controls.

Following infection, an increasing frequency of dogs displayed a positive serology to *Leishmania*. Ninety days after infection 37.1% (13/35) of dogs were positive for anti-*Leishmania* IgG that increased to 62.9% (22/35) after 180 days. Two hundred and seventy days after infection, this frequency increased to 77.4% (24/35) and finally after 450 days of infection 85.14% (18/21) were positive for IgG anti-*Leishmania* (Figure 4A). Interestingly, we observed increased levels of IgG2 subclass and not IgG1 in experimentally infected dogs (Figures 4B and C). There was a considerable titer variation among dogs, and even after more than one year of infection, eight dogs did not show detectable antibody titers.

### Correlations between Parasite Load, Clinical Score and Levels of IgG

**Figure 5:**
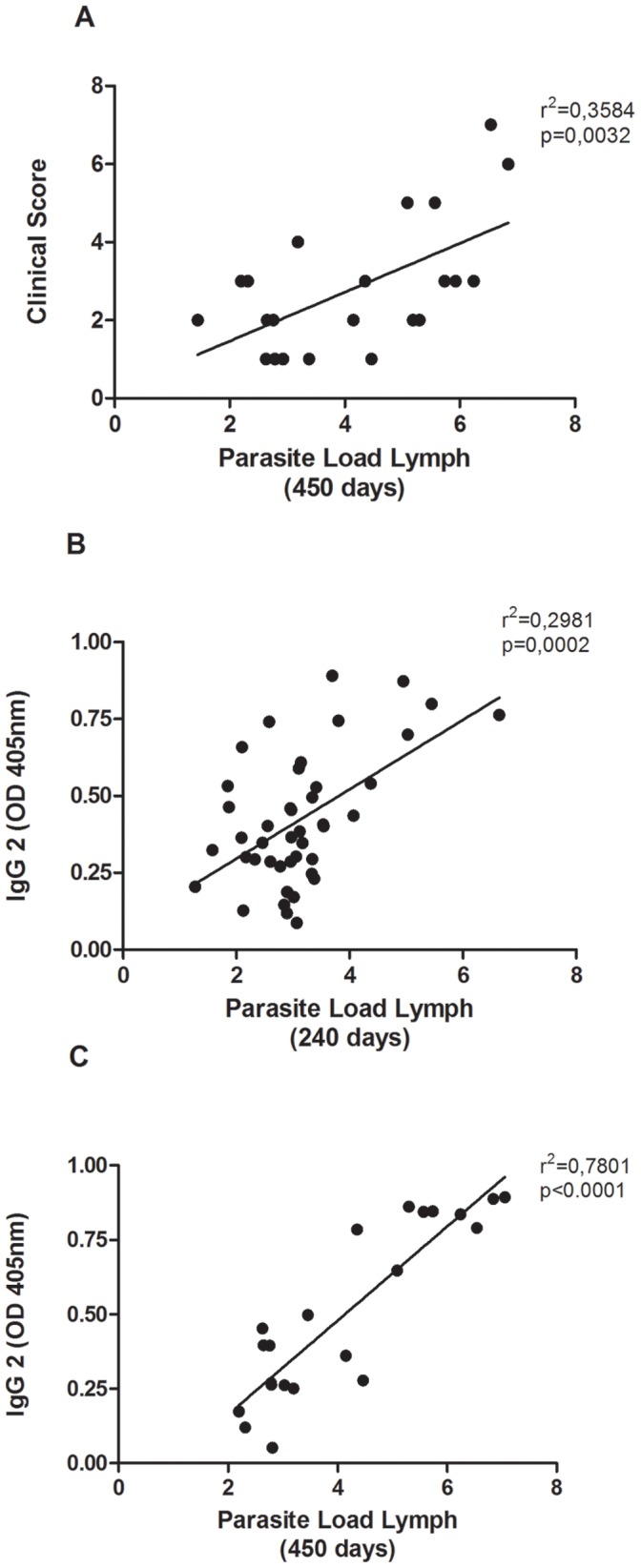
Correlation between parasite load in popliteal lymph nodes with clinical score in experimentally infected dogs. Correlation of Spearman between parasite load at popliteal lymph nodes with clinical score after 450 days of infection (A) and levels of IgG2 240 and 450 days after infection (B and C).

There was a significant positive correlation (p<0.01 and r^2^ = 0.53) between parasite load and clinical score 450 days after infection (Figure 5A). A similar correlation was also observed between parasite load and levels of anti-*Leishmania* IgG or IgG2 where the number of parasites correlates with IgG or IgG2 titers 240 and 450 days after infection (Figures 5B and C).

### Cytokine Response

**Figure 6:**
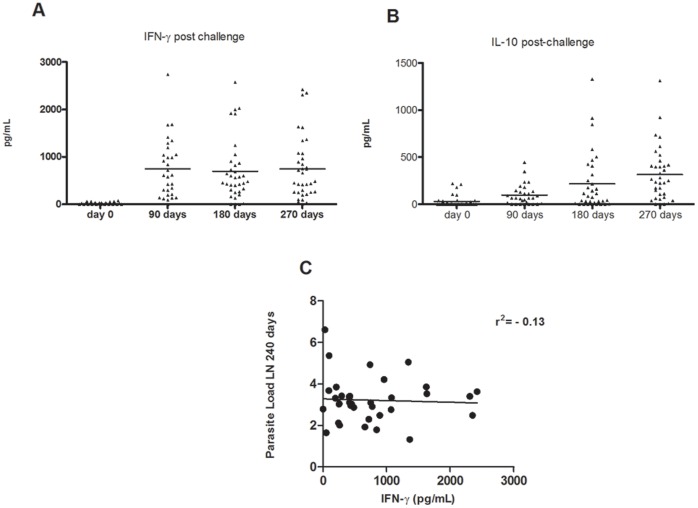
Levels of IFN-γ and IL-10 in supernatants of PBMC from experimentally infected dogs. Dogs were infected with *Leishmania infantum-chagasi* plus five pairs of *Lutzomyia longipalpis* salivary glands. PBMC were obtained at days zero, 90, 180 and 270 post infection re-stimulated *in vitro* with *Leishmania infantum-chagasi* and the supernatant collected after 48 and 72 hours for determination of IFN-γ (A) and IL-10 (B) by ELISA, respectively. (C) Spearman correlation between IFN-γ levels and parasite load at 240 days after infection.

PBMC from infected dogs were obtained at different time points after infection (zero, 90, 180 and 270 days post-infection) and were re-stimulated *in vitro* with *Leishmania infantum* promastigotes. After 48 and 72 hours of culture, supernatants were collected and the levels of IFN-γ and IL-10 production determined. At 90, 180 and 270 days post-infection there was an increase in IFN-γ production (Figure 6A) as well as IL-10 (Figure 6B). Interestingly, IFN-γ levels were maintained during infection whereas IL-10 concentration showed a progressive increase. However, the ratio of IFN-γ/IL-10 was constant during the infection with predominance of IFN-γ production (data not shown). There was no correlation between IFN-γ levels and parasite load 240 days after infection (Figure 6C), suggesting that the levels of this cytokine are not enough to control parasite growth.

## Discussion

To date, there is no strategy able to treat or control canine visceral leishmaniasis. The establishment of a canine model will bring opportunities to study new vaccine candidates as well as different drugs for CVL treatment. In this study, we described an experimental approach where dogs were infected intradermally with *Leishmania infantum* combined with saliva from *Lutzomyia longipalpis*. All dogs became infected and several developed symptomatic infection and for this reason were euthanized 240 days after infection. The clinical scores displayed by these animals were similar to those observed in naturally infected dogs, suggesting that injection of parasites plus saliva could be an important experimental approach, since the ability to perform natural transmission by sand flies is restricted to few laboratories. Although the number of parasites as well as the amount of glands used in this study is higher compared to previous work in the literature, our objective in the future is to test potential vaccine candidates using salivary proteins reasserting the importance to establish a model in dogs that includes vector saliva always present during transmission in endemic areas.

The majority of animals were asymptomatic surviving more than one year. This is also observed in endemic areas, where infected dogs remain asymptomatic or show transient disease after infection [26].

In the group of experimentally infected dogs living under controlled conditions, we also observed a range of different clinical manifestations and parasite load from popliteal lymph nodes and PBMC, suggesting that factors inherent to the host contribute to the variability of clinical aspects or outcome of the infection [27], [28], [29]. Dogs displaying severe symptoms of CVL showed high parasite load and they were more infective to sand flies confirmed by xenodiagnosis where around 21% of the dogs were able to transmit parasites to uninfected sand flies. In fact, we found out that a minimum of 3.35×10^5^ parasites in the lymph nodes and 3.34×10^2^ in PBMC was required to result in a positive xenodiagnosis.

Interestingly some of the experimentally infected dogs showed a positive serology but they were non-infective to sand flies. This information is of highest importance, especially in endemic areas where culling of dogs that present a positive anti-*Leishmania* serology is obligatory. It could be used to improve evaluation of dogs present in endemic areas considering the selection of those that are more infective and responsible for the constant VL transmission. Similar results were found by Travi *et al.* [17] where only poly-symptomatic dogs were infective, while the majority of infected dogs were non-infective to laboratory-reared sand flies [17]. Other studies have already shown that infectivity to sand flies correlates with severity of disease [30], [31], [32].

Interestingly, we observed that the skin of naturally infected dogs showed significantly lesser parasites than those experimentally infected. The efficiency of vector-mediated transmission of *Leishmania infantum* in endemic areas, mainly in dogs, has not been well established. Verçosa *et al*. studying the transmissibility of naturally infected dogs in a CVL endemic area observed that the presence of parasites in the skin is considered important for transmission of parasites to the sand fly [33]. Our results also pointed out the importance of parasite load to result in a positive xenodiagnosis, but in our findings these dogs also displayed higher clinical score than those with a negative xenodiagnosis. On the other hand, Travi *et al.* [32] observed that there is no correlation between presence of parasite in the skin and potential transmission to the vector.

We observed that even though all dogs had parasites detected in the lymph nodes, some (10/35) still maintained a negative serology, suggesting that this parameter is not appropriate for diagnosis of CVL while the detection of parasites seems to be more accurate. Similar findings were described with naturally infected dogs where 11% of the animals showing positive splenic cultures had a negative serology for *Leishmania* [34]. However, we also confirm a significant positive correlation between levels of IgG or IgG2 and parasite load in the lymph node of infected dogs. We also observed a positive correlation between clinical score and parasite load that corroborate with the same observation previously described [17], [34]. Although in the mouse model, IgG1 and IgG2 levels are used as a marker of Th2 and Th1, respectively, this correlation is not clear in dogs. Several different studies showed an increase in the levels of IgG2 anti-*Leishmania* in infected dogs [35], [36], [37], [38], [39], [40], whereas the presence of IgG1 is more controversial [33], [39], [40]. Different studies have associated high levels of IgG2 with asymptomatic disease and high IgG1 concentrations with active disease [36], [37], [40]. In our study, we observed an increase in the concentration of IgG2, very similar to total IgG while IgG1 levels showed a greater variation during the infection. Similar results were previously described comparing infection of dogs through injection of parasites intradermally or intravenously and found levels of IgG2, independent of the route used for infection [17].

Detection of cytokine production in CVL is controversial. Protective responses in dogs have been associated with the lack of clinical symptoms, low levels of anti-*Leishmania* antibodies, parasite load and a positive DTH. Therefore, a cellular immune response in CVL is associated with activation of Th1 response, with IFN-γ, IL-2 and TNF-α production [28], [41]. On the other hand, Reis *et al.* [42] showed that active disease is characterized by a marked humoral immune response. The clinical symptoms that correlated with parasite load in different tissues are associated with a specific immunosupression. In these animals, there is a mixed Th1/Th2 response [41], [43]. In our study we observed that at day 90 post-infection, we detected IFN-γ following PBMC stimulation with *Leishmania* promastigotes and the levels did not change during infection. Many reports have shown the importance of IFN-γ increase and IL-10 decrease to obtain a protective response against *Leishmania* in different experimental protocols to test vaccine candidates [26], [44], [45], [46], [47], [48]. In dogs, low parasite load in the lymph nodes was also associated with a high production of IFN-γ and TNF-α [26]. Travi *et al.* [17] noticed that expression and production of IFN-γ was detected earlier in dogs intradermally infected than those infected by intravenous route although the peak of IFN-γ production occurred six months post-infection independently of the route of infection. They also observed that most symptomatic dogs produced high levels of IFN-γ at the early stages of infection and the proportion of animals producing this cytokine increased over time. Our results also suggested that IFN-γ was not sufficient to prevent disease and it could not be considered a marker of resistance. We also observed a high production of IL-10 ninety days after infection with a slight increase through time, but the levels were not significantly different between the time points evaluated. We did not find any correlation of IL-10 levels with parasite load in the lymph nodes or clinical symptoms. However, the frequency of dogs producing high levels of IL-10 increased after 270 days of infection, suggesting that the production of this cytokine might contribute to infection severity.

Interestingly, few reports in the literature have demonstrated the role of saliva in the immunopathogenesis of infection by Leishmania, mainly in different models of Visceral Leishmaniasis. Its role is well demonstrated in the establishment of Cutaneous Leishmaniasis infection, mostly using *L. major* [8], [9], [10], [11], [12], [13], [14], [15]. However, our results agree with the findings observed in naturally infected dogs and autochtonous infected dogs, where increased IL-10 expression is associated with disease progression [49].

One important aspect of our experimental model is the inoculation of saliva together with parasites. Although *L. infantum* infection can be achieved without co-inoculation with saliva, it is extremely relevant to recognize that saliva is always present during natural transmission through the bite of infected sand flies in endemic areas where ultimately a future vaccine or control measure would be implemented. Saliva has an important role in parasite establishment in the host demonstrated in different animal models [19]. Recently, the possibility to use salivary proteins from sand flies as components of a vaccine candidate has been demonstrated in dogs. Immunization with LJM17 and LJL143, two salivary proteins from *Lutzomyia longipalpis*, were able to induce an intense immune response with a high production of IFN-γ and low levels of IL-10 and TGF-β eliciting a DTH response after challenge of uninfected sand flies with an adverse effect to parasite infection *in vitro* [50]. Indeed, Roatt *et al.* tested a vaccine called LBSap composed of *L. brazilensis* antigens plus saponin using intradermal challenge with *L.infantum* and saliva of *Lutzomyia longipalpis.* Therefore, this experimental model will allow us to test the efficacy of salivary proteins based vaccine candidates. More importantly, it also brought relevant new information that could help in a more adequate evaluation of dogs residing in a VL endemic area that could be more transmissible to sand flies serving as potential reservoirs.
